# The Effects of Laser Parameters and the Ablation Mechanism in Laser Ablation of C/SiC Composite

**DOI:** 10.3390/ma12193076

**Published:** 2019-09-20

**Authors:** Sining Pan, Qingyu Li, Zhaokun Xian, Nanguang Su, Fanzheng Zeng

**Affiliations:** 1 College of Mechanic and Electronic Engineering, Hezhou University, Hezhou 542899, China; 2 Institute of Mechanics, Chinese Academy of Sciences, Beijing 100190, China; 3 School of Engineering Science, University of Chinese Academy of Sciences, Beijing 100049, China

**Keywords:** Laser ablation, C/SiC composite, Surface morphology, Ablation mechanism, Scanning electron microscopy (SEM)

## Abstract

The effects of laser parameters and the ablation mechanism in laser ablation of a carbon fiber reinforced silicon carbide (C/SiC) composite are investigated in the present study. Six different power densities are provided, as well as six levels of pulse numbers, and then ablation experiments are conducted for the C/SiC composite, induced by a pulsed laser. Based on the experimental results, the characteristics of surface morphology and ablation behavior are discussed. It is revealed that the surface morphology of the C/SiC composite under laser irradiation usually includes three regions: the center region, the transition region, and the border region. With the increase of laser power density, the ablation of the center region becomes severe, surface cracks occur, and more spherical SiC particles are found in the transition region. As for scenarios involving multiple pulses, the damage occurs in the center region at low power density limits, within the first two layers below the surface. However, if the power density is relatively high, an ablation pit occurs in the center region when the pulse number is larger than 50. Meanwhile, the transition region and the border region diminish with increase of the pulse number. It is noted that both the power density and pulse number have noticeable effects on surface morphology and ablation behavior during laser ablation, which is helpful for material design and performance evaluation of C/SiC composites.

## 1. Introduction

Advanced composite materials (and more specifically carbon fiber reinforced polymer (CFRP)) are important for various applications, such as structural applications, environmental barrier coatings, and nuclear applications. This is because of their excellent properties, including having low density, extraordinary high-temperature strength, and high resistance to thermal shocks and ablation [1,2]. With the increase of service temperature, more carbon-fiber-reinforced composites have been developed.

As a typical and promising material system, carbon-fiber-reinforced silicon carbide (C/SiC) composites possess many attractive characteristics, such as high fracture toughness, excellent high-temperature strength, good thermal shock and ablation resistance, as well as high reliability [3,4,5,6]. Therefore, they have been widely used as combustion chambers, thruster components, and nozzles for rocket engines and heat-exchanger parts, among others [7,8,9,10]. Because of the urgent need for high temperature materials for high performance thermal protection system (TPS) applications, C/SiC composites have important application prospects, which are worthy of intensive study. SiC can be processed by spark plasma sintering (SPS), and can be structurally modified by ion irradiation. Moreover, C/SiC composites have temperature limits under long term operation in an oxidizing environment caused by the matrix transition from passive to active oxidation, with the oxidation rate increasing quickly with the temperature [3,11,12]. These strategic applications require C/SiC composites to have excellent thermal ablation performance, in addition to high specific strength and fracture toughness [13,14], which could directly improve the dimensional stability and thermal stress distribution of the materials at high temperatures. Therefore, it is essential to investigate the thermal ablation properties of C/SiC composites as important design factors.

The main experimental methods for the thermal ablation process for the C/SiC composite are oxyacetylene flame [15,16], plasma arc [17,18], and wind tunnel tests [19]. In past years, a large number of research studies have focused on the ablation-resistant properties of C/SiC composites, and great achievements have been made [20,21]. Nowadays, laser beams are promising tools for C/SiC composite inveatigation and processing, indicating that laser ablation is a facile and reliable method for evaluating ablation resistance of materials [22,23,24]. In addition, lasers are considered an ideal heat source for ablation experiments due to having high spatial and temporal controllability [25]. The evaluation of ablation resistance by lasers can provide more knowledge about the usability of C/SiC composites, and can help develop protection against laser irradiation.

In recent years, the ablation behavior of C/SiC composites under laser irradiation has been widely studied. Tong et al. [23,26] used a pulsed laser to investigate the ablation behavior and mechanisms of a C/SiC composite. The linear ablation rate was discussed, and three ablation regions were identified on the ablated surface. Wu et al. [27] investigated the ablation behaviors of C/SiC laminates subject to continuous wave, long duration pulsed wave, and short duration pulsed wave lasers. Based on the experimental and calculated results, distinctive ablation phenomena were observed. Liu et al. [28] experimentally and numerically studied the thermomechanical responses of C/SiC composites under continuous wave laser irradiation. In particular, the ablation damages induced by the thermal loading were studied, and the effects of laser power on the ablation behavior were discussed. Wang et al. [29] investigated continuous wave laser ablation behavior of C/SiC composites subjected to supersonic airflow and a static air environment. The microstructure of the ablation region was measured. The effects of supersonic airflow on the ablation morphology and ablation rates of the C/SiC composite were analyzed. Although a large number of research results have been reported, the effects of laser parameters on the ablation behavior during the laser ablation process have seldom been discussed, and the laser ablation mechanisms of C/SiC composites are still unclear. In addition, detailed research studies on the formation of ablated cracks are still needed in order to provide additional information for material properties, such as material strength and toughness [30,31].

In the present paper, a pulsed laser is used to evaluate the ablation resistance of a C/SiC composite. The effects of laser parameters on the laser ablation, especially power density and pulse number, are studied experimentally. The ablation microstructure under different laser parameters is observed, and the laser ablation behavior and mechanisms of the composite are investigated.

## 2. Experimental Description

### 2.1. Experimental Setup

**Figure 1:**
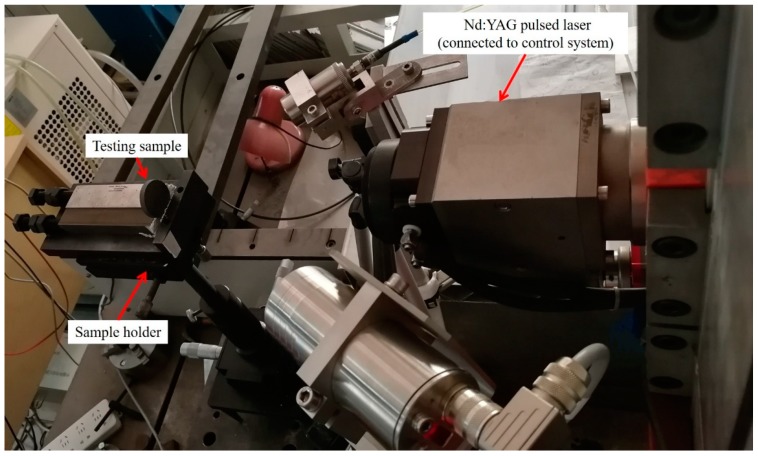
Experimental assembly of the laser ablation process.

The experimental assembly is presented in Figure 1. An experimental system was designed based on a IQL-10 Nd/YAG millisecond pulsed laser with a wavelength of 1064 nm, which mainly served as the heat source in the air. The pulsed laser was connected to the control system, and served with the following parameters: frequency 0.5 Hz, pulse width 1 ms, laser spot diameter approximately 2 mm. The testing conditions for the laser ablation process were that same as is shown in Table 1. In order to investigate the effect of power density, six levels of single pulse energy were selected, ranging from 1.5 to 3.8 J. The effect of laser–material interaction and the limit capability of the pulsed laser were considered during the parameter selection. Once the pulse energy was selected, the corresponding peak power and peak power density were calculated according to the pulse width and laser spot diameter.

### 2.2. Specimen Preparation

**Figure 2:**
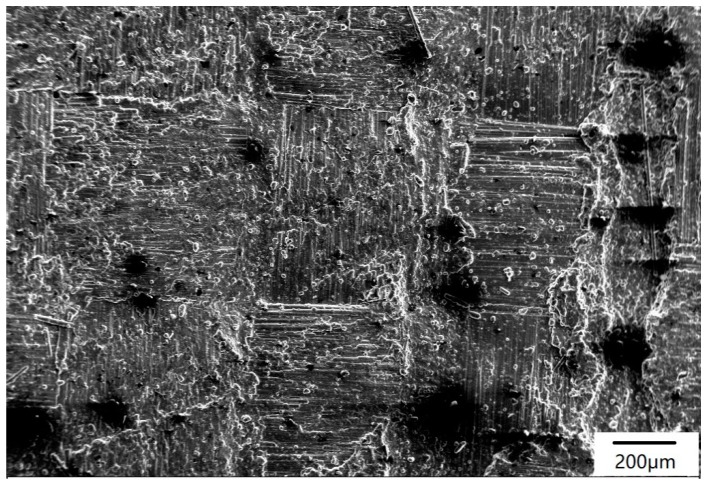
Surface morphology of the C/SiC composite.

A two-dimensional C/SiC composite was prepared by chemical vapor infiltration (CVI) and polymer impregnation pyrolysis (PIP) methods. The fiber volume fraction was approxiamtely 40% in the final composite. Low pressure CVI was employed to deposit the pyrolytic carbon layer and the silicon carbide matrix. The interfacial layer of pyrolytic carbon was prepared by using methane as the carbon source gas and argon as the dilution gas. The deposition temperature was maintained at 900–1000 °C, and the deposition time was 15–25 h. The parameters of the PIP process were as follows: impregnation time was 2–4 h, impregnation pressure was 1.8–2.0 MPa, and pyrolysis temperature was 1000–1300 °C. The SiC coating was prepared by chemical vapor deposition (CVD) on the sample surface. The CVD-SiC coating was prepared by using trichloromethylsilane as the reaction precursor, argon as the dilution gas, and hydrogen as the carrier gas, with a deposition temperature of 1000–1200 °C. The specimen had a diameter of 30 mm and a thickness of 5 mm. The typical surface morphology of the C/SiC composite studied in this paper is shown in Figure 2. The morphologies of C/SiC composites before and after ablation testing were analyzed using a JSM-6700 (JEOL, Tokya, Japan) scanning electron microscope (SEM).

## 3. Results and Discussion

### 3.1. Laser Ablation Morphology

#### 3.1.1. The Effect of Power Density

In order to investigate the effect of power density on the ablation morphology, the experiments were implemented using a single laser pulse. When the sample is exposed to the laser beam, laser energy is absorbed by the sample and dissipated into thermal energy quickly [27]. As a result, the physical and chemical actions occur, such as pyrolysis and ablation, inducing thermal damage in the C/SiC composite [32].

**Figure 3:**
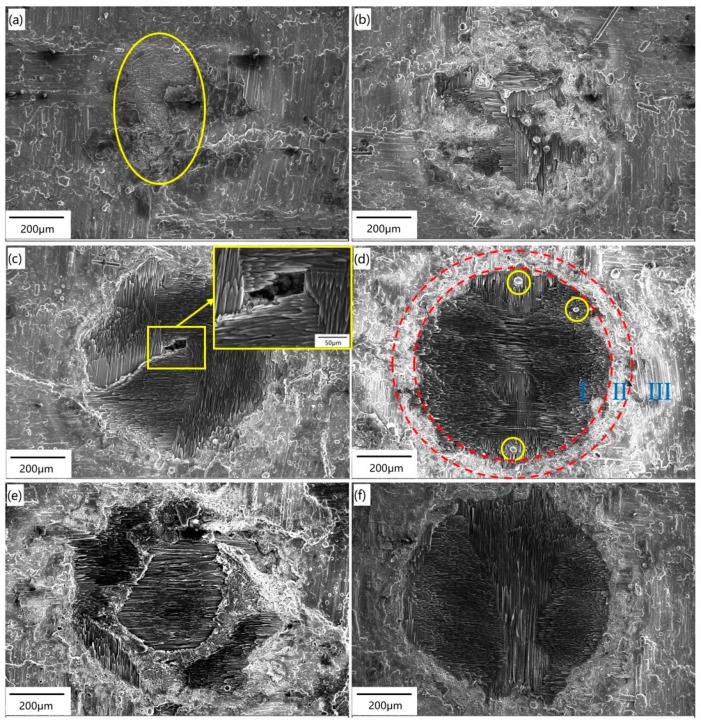
Surface morphology of the C/SiC composite irradiated by a single pulse. (**a**) Condition A; (**b**) Condition B; (**c**) Condition C; (**d**) Condition D; (**e**) Condition E; (**f**) Condition F.

Figure 3 shows the surface morphology of the C/SiC composite irradiated by different power densities, as listed in Table 1. Figure 3a shows that when the power density is relatively low, the ablation of the SiC coating on the surface occurs, and the SiC matrix in the first layer is exposed to the laser beam. Therefore, decomposition and oxidation of the SiC matrix can be expected, which is depicted with a yellow line in Figure 3a. As the laser energy increases (see Figure 3b), the surface coating is ablated due to the high temperature in the center of the laser spot. At the same time, the SiC matrix in the first layer is also decomposed at high temperatures, therefore, the non-ablated carbon fibers of the first layer are exposed. It is likely that the temperature increase caused by laser irradiation under Condition B reaches the decomposition temperature of the SiC matrix, but does not reach the melting point of the carbon fibers. Therefore, the SiC matrix is ablated, while the carbon fibers are retained and exposed on the sample surface. There are several large spherical particles in the ablation center, which is the oxidation product of SiC. In addition, a large number of small spherical particles are also distributed at the ablation edge of the SiC coating. 

As the laser power continues to increase (see Figure 3c), the boundary between the irradiated region and non-irradiated region is clearly visible. The surface coating, the SiC matrix, and the carbon fibers of the first layer are all ablated. Not only are the surface coating and SiC matrix of the first layer sublimated completely, but also some carbon fibers in the first layer are sublimated. The sublimation temperatures of SiC and carbon fibers are 2700 °C and 3550 °C, respectively [20], indicating that the temperature in the center region is above 3550 °C. From the graph insert in Figure 3c, it can be seen that since the center of laser spot is located in the pore of the first layer, the laser beam is self-trapped in that location, causing multiple reflections and energy absorption. Therefore, the second layer of the SiC matrix is oxidized, and the first layer of carbon fibers is exposed around the pore.

When the power density is increased to 9.55 × 10^4^ W·cm^−2^, three different regions can be distinguished on the ablated surface (depicted by red dotted lines in Figure 3d): region I, the ablation center with a needle-like structure; region II, the transition region with many spherical particles; and region III, the ablation edge covered by a white glassy layer. This is similar to the results in a previous study [26]. Within the boundary between the center region and the transition region, there are three white spherical particles (marked by yellow circles). Most of the carbon fibers distributed horizontally in the first layer are ablated, while the carbon fibers distributed vertically in the first layer are exposed.

As shown in Figure 3e, the ablation damage becomes more severe as the laser energy increases. The ablated areas include the surface coating, the SiC matrix, the carbon fibers of the first layer, the SiC matrix of the second layer, and the carbon fibers of the second layer, which are partially located in the spot center. The partially ablated carbon fibers of the second layer are exposed on the sample surface. The laser energy is higher the closer it is to the center of the beam. Additionally, the higher the sample temperature is after absorbing laser energy, the more severe the ablation. As for Condition F (see in Figure 3f), the ablation of carbon fibers in the second layer is further increased and the fibers are exposed to the sample surface after testing. With the increase of power density, the boundary of the three regions becomes more obvious. Furthermore, the ablation of the center region increases gradually, and more spherical SiC particles are deposited in the transition region. 

**Figure 4:**
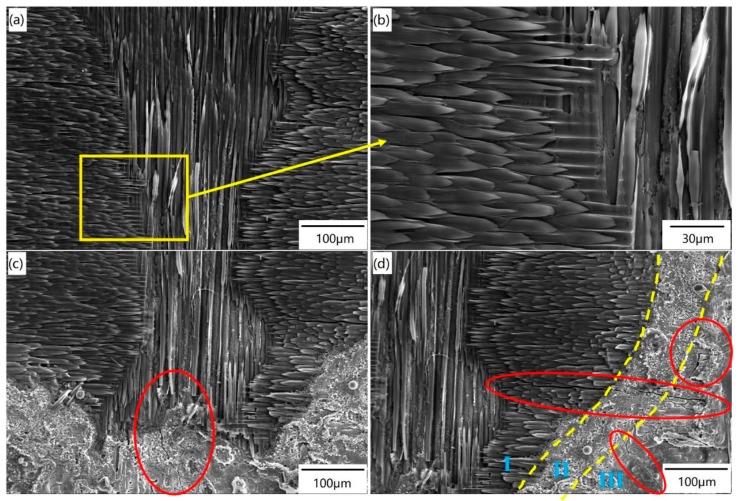
Different areas on the irradiated surface under Condition F: (**a**,**b**) center region; (**c**) transition region; (**d**) border region.

Figure 4 shows the SEM photograph of different areas of the irradiated surface under Condition F. Obviously, it can be seen in Figure 4a that the center region is ablated heavily due to the highly focused laser beam. Once the SiC matrix is sublimated completely, the carbon fibers are fully exposed. The carbon fibers are ablated, breaking off in the center region, as shown in Figure 4b with larger magnification. The carbon fibers located in the center region are ablated into sharp “needle-like” shapes, which are usually observed in C/SiC composites during ablation by oxidation. The middle part of Figure 4b shows the remaining SiC matrix between the naked fiber bundles. In the transition region (Figure 4c), the SiC matrix is not sublimated completely and has a spherical structure. A surface microcrack takes place due to the severe thermal shock during testing, which is located at the boundary between the center region and transition region, as depicted with a red line. This crack is caused by thermal shock because the peak stresses arise in the cooling stage after the laser beam is removed, when the maximum temperature gradients are developed between the surface and the inside [28]. As shown in Figure 4d, the ablation in the border region is lighter than that in the center region. As marked with red lines in Figure 4d, there are two surface cracks located in the border region, and a long crack starts from the fiber bundles in the center region, propagating into the border region. When the power density is relatively low, the temperature difference between the center and the edge of the laser spot is small, and cracks do not occur easily. If the power density is increased high enough, there will be a large temperature difference between the center and the edge of the laser spot. Once the laser beam is removed, the sample surface enters into a rapid cooling stage, which is equivalent to bearing the thermal shock loading. Therefore, thermal shock cracks tend to form.

#### 3.1.2. The Effect of Pulse Number

Furthermore, in order to assess the effect of pulse number, a study is carried out based on the same laser power density, considering two levels: Condition A and Condition D.

**Figure 5:**
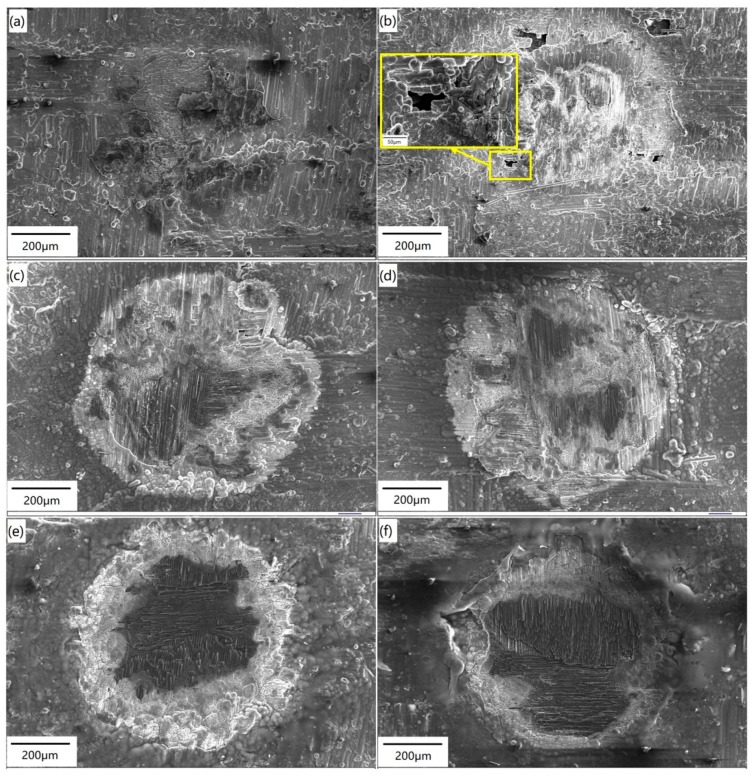
Surface morphology of the C/SiC composite irradiated under Condition A for different pulse numbers: (**a**) 1 pulse; (**b**) 2 pulses; (**c**) 5 pulses; (**d**) 10 pulses; (**e**) 50 pulses; (**f**) 100 pulses.

When the power density is relatively low, as in Figure 5a, the ablation of the surface coating and the oxidation of the SiC matrix in the first layer occur after ablation for only 1 pulse. After ablation for 2 pulses, the boundary between the laser irradiated area and non-irradiated area becomes obvious, with a diameter of about 600 μm, as shown in Figure 5b. The ablation of the SiC matrix in the first layer occurs, but almost no carbon fibers are exposed to the laser directly. In addition, there are several ablated pores located within the irradiated area, and surface cracks can be found around these ablated pores, as shown in the insert graph in Figure 5b. After ablation for 5 pulses (see Figure 5c; the ablation time is 10 s), the SiC matrix of the first layer at the spot center is ablated completely. The diameter and depth of the ablation area are about 700 μm and 200 μm, respectively. The carbon fibers of the first layer are exposed to the laser beam and ablated until they break off. The SiC matrix without naked carbon fibers is melted at high temperature, and then re-solidified into a large number of white spherical particles. A large quantity of fine cracks are distributed on the surface within the laser spot. 

After 10 pulses have been applied (see in Figure 5d), the surface morphology of the laser spot is similar to that after 5 pulses, but the ablation is more severe than in the previous description. The diameter and depth of the ablation area are nearly 750 μm and 400 μm, respectively. In Figure 5e, the carbon fibers with vertical distribution in the first layer have been completely ablated, while the horizontal ones have been partially ablated, breaking off after 50 pulses. The diameter and depth of the ablation area are nearly 750 μm and 600 μm, respectively. There are three distinct regions on the sample surface: the center region, the transition region, and the border region. A lot of surface cracks are found in the transition region and the border region. After 100 pulses, as shown in Figure 5f, the carbon fibers with vertical distribution in the first layer have been completely ablated, and the tips of the ablated fibers are needle-shaped. The carbon fibers of the first layer, which are distributed horizontally, are ablated until they break off. The SiC matrix of the second layer, which is exposed to the laser beam, is also oxidized. The diameter and depth of the ablation area are about 780 μm and 800 μm, respectively. Due to the long ablation time with the pulsed laser, the products formed during the repeated ablation–cooling process continuously flow to the sample edge, resulting in obvious protrusions in the border region. From the above discussion, it can be inferred that when the power density is relatively low, even after being ablated for 100 pulses, the ablation damage is still limited in the first two layer under the sample surface. 

**Figure 6:**
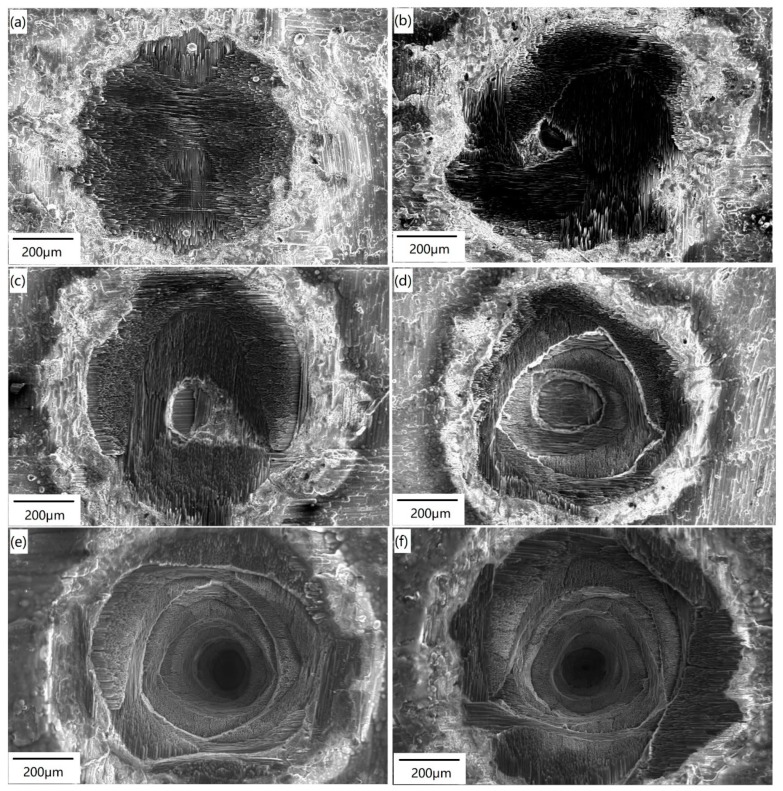
Surface morphology of the C/SiC composite irradiated under Condition D with different pulse numbers: (**a**) 1 pulse; (**b**) 2 pulses; (**c**) 5 pulses; (**d**) 10 pulses; (**e**) 50 pulses; (**f**) 100 pulses.

Figure 6 shows the morphologies of the ablation surface after multiple pulses at relatively high power density. In Figure 6a, the surface presents a typical three-region morphology, as described above. The carbon fibers with horizontal distribution in the first layer are almost ablated, while the vertically ones are exposed. As illustrated in Figure 6b, after 2 pulses, since the spot center is located in the gap between two orthogonal fiber bundles in the first layer, almost all of the laser energy is absorbed by the material near the gap. Therefore, high-temperature ablation occurs in the atmosphere. The carbon fibers of the first layer, accompanied with the SiC matrix of the second layer near the gap, are all ablated, and the exposed carbon fibers of the second layer are oxidized. When irradiated by 5 pulses (as shown in Figure 6c), not only are ablated completely the matrix and carbon fibers of the first layer, but the matrix and carbon fibers distributed vertically in the second layer are also, leading to the exposure of the carbon fibers with horizontal distribution in the second layer. In the center region, the ablation area of the first layer is larger than that in the second layer, showing a bowl-shaped structure. 

With the increase in pulse number, it can be noticed that the number of ablated layers increases gradually. After 10 pulses, as in Figure 6d, the area of complete ablation includes the matrix and carbon fibers from the first to the third layers below the sample surface. The exposed carbon fibers with horizontal distribution in the fourth layer are partially ablated. In the center region, the ablation area of each layer decreases gradually from the surface to the bottom. This is because the laser energy is spatially distributed in the form of the Gauss function. The closer the laser energy is to the spot center, the higher the laser energy is, and the heavier the ablation. This tendency becomes more obvious with the increase of the pulse number, which can be seen in Figure 6e,f. The above observation suggests that when the laser power density is relatively high, the number of ablated layers within the spot center increases with the increase of pulse number. In addition, the transition region and border region become smaller, and the ablation products in the border region become more obvious with the increase of pulse number.

### 3.2. Laser Ablation Mechanism

As for the C/SiC composite, the laser ablation is a complex process, which mainly consists of oxidation, evaporation, and thermal decomposition [33]. During the laser ablation testing, the energy of the pulse laser is absorbed by the sample, depending on several characteristic parameters, such as the absorption coefficient, the heat conduction width, and the heat conduction depth [34]. Along the heat conduction width and conduction depth, the input energy decreases rapidly from the original value. Therefore, the temperature distribution caused by the laser irradiation is strongly influenced by incident energy. Generally, the higher the power density, the larger the heat conduction width, and the greater the heat penetration depth [26].

When the composite is irradiated by a pulsed laser, the center region is instantly heated to an extremely high temperature, which is greater than 3500 °C. The SiC matrix reaches the decomposition and sublimation temperatures, and a hot mixture consisting of gases and vapors is formed, caused by the low thermally stable temperature [35]. At the same time, the carbon fibers may also reach their sublimation temperature to form a carbon vapor. For the ablation temperature above 3500 °C, all of the following reactions can possibly proceed:

(1) SiC(s)→Si(g)+C(g)

*SiC*(*s*) → *SiC*(*g*) (2)

*SiC*(*s*) + *O*_2_(*g*) → *SiO*_2_(*l*) + *CO*(*g*) (3)

*SiC*(*s*) + *O*_2_(*g*) → *SiO*_2_(*g*) + *CO*(*g*) (4)

*SiC*(*s*) + *O*_2_(*g*) → *SiO*(*g*) + *CO*(*g*) (5)

*SiC*(*l*) → *SiC*(*g*) (6)

*SiC*(*s*) + *SiO*_2_(*l*) → *SiO*(*g*) + *CO*_2_(*g*) (7)

*SiC*(*s*) + *CO*_2_(*g*) → *SiO*_2_(*l*) + *CO*(*g*) (8)

*C*(*s*) → *C*(*g*) (9)

*C*(*s*) + *O*_2_(*g*) → *CO*_2_(*g*) (10)

*C*(*s*) + *O*_2_(*g*) → *CO*(*g*) (11)

*C*(*s*) + *CO*_2_(*g*) → *CO*(*g*) (12)

Since the ablation testing is conducted in open air, the SiC matrix is oxidized after ablation due to its poor oxidation resistance at higher temperature, according to Equations (1)–(8). Since the vapor pressure of SiO_2_ at the testing temperature is very high [36], SiO_2_ produced by the oxidation reactions could directly gasify (the boiling point of SiO_2_ is 2230 °C), according to Equation (6) [37]. As it is exposed to the laser, the center region of the C/SiC composite experiences the highest temperature and is severely ablated. The decomposition of the SiC matrix consumes thermal energy, which is beneficial in reducing ablation. Carbon fibers are easily oxidized, and they obey the oxidation reactions (Equations (9)–12)). Since the carbon fibers are more stable than the SiC matrix, the carbon fibers protrude on the ablation surface, forming a needle-like microstructure.

As stated above, in the center region, the heat energy induced by the pulsed laser penetrates into the sample. The penetration depth is related to the heat energy on the surface. Since the corresponding temperature decreases with the increasing penetration depth, the ablation of the carbon fibers is alleviated gradually, and finally ceases along the heat conduction direction. However, the temperature is still high enough for decomposition of the SiC matrix. As a result, many grooves can be found among the carbon fibers without the SiC matrix, as shown in Figure 4b. With the increase of power density, the ablation of the center region gradually becomes more severe. If the laser power density is high enough, a large temperature difference will be induced between the center and the edge of the laser spot, which is related to the spatial distribution of the Gaussian laser. Once the laser beam is removed, the sample surface enters into a rapid cooling stage, causing the formation of thermal shock cracks. 

Furthermore, when the power density is relatively high, the number of ablated layers in the center region increases with the increase of the pulse number. This phenomenon can be explained in terms of effective area for energy absorption. The sample surface is relatively smooth before the ablation test. Once the input energy is large enough, ablation will occur in the center region, and therefore, the ablated matrix and carbon fibers are exposed. As a result, the surface roughness increases, and the effective area for energy absorption from the pulsed laser also increases. When the next pulse continues to irradiate in the region, more laser energy will be absorbed by the ablated surface, and the region with high temperature will be enlarged, thus producing more severe ablation than the previous pulse. With the continuous action of the pulsed laser, an avalanche effect is formed in the center region. The number of ablation layers increases with the increase of pulse number, forming more obvious ablation pits, as shown in Figure 6e,f.

In the transition ablation region, the heat energy and the corresponding penetration depth are much lower than that in the center region. Thus, the oxidation of carbon fibers and decomposition of the SiC matrix are less severe. Generally, the temperature is not high enough for the decomposition and sublimation of the SiC matrix. The gas mixture of Si, C, and SiC generated from the ablated center (Equations (1), (2), and (9)) is cooled down and reforms into spherical SiC particles. However, the temperature is considered to be high enough for the volatilization of SiO_2_ (~2230 °C), so almost no SiO_2_ is found in the transition region. In Figure 3, it is noted that the boundary of the three regions becomes obvious with the increase of laser power density, and more spherical SiC particles are deposited in the transition region. When the power density is relatively high, the transition region diminishes with the increase of the pulse number, as shown in Figure 6.

At the border region, the conducted temperature is the lowest among the three regions, which only causes the oxidation of the C/SiC composite. The border region around the ablated pit is covered by a viscous silica glass layer, which mainly consists of the oxidation product SiO_2_ from the SiC matrix. As oxygen can barely infiltrate the SiO_2_ layer, the fibers below this glassy layer remain less oxidized. The oxide products in the SiC matrix are SiO_2_ in the conventional oxidation reaction (Equations (3) and (4)) and SiO in the active reaction (Equation (5)) [13]. The SiO_2_ produced in the center region evaporates and condenses at the border region. At the same time, SiO combines with oxygen and condenses into a silica-rich glassy layer caused by the lower temperature in this region. Similar to the transition region, with the increase of pulse number, the border region diminishes gradually, and the ablation effects in the border region become more obvious.

**Figure 7:**
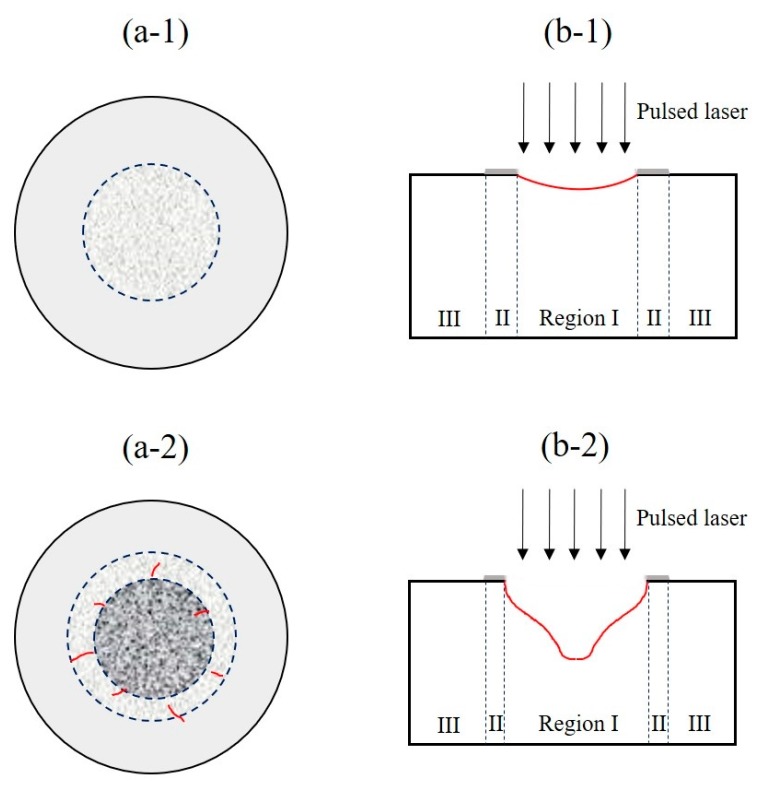
Laser ablation model of the C/SiC composite. The effect of laser power density (single pulse): (**a-1**) low power density, (**a-2**) high power density. The effect of pulse number (multiple pulses): (**b-1**) low power density, (**b-2**) high power density.

In order to illustrate the effects of power density and pulse number in the laser ablation of the C/SiC composite, an ablation model based on the above results is proposed, which is represented schematically in Figure 7. As described above, it is revealed in Figure 7a-1,a-2 that the C/SiC composite under laser irradiation mainly consists of three regions at high power density, while the boundary seems obscure at relatively low power density. In center region I, the compound formed by gases and vapors is generated by the sublimation of the SiC matrix and the carbon fibers. The temperature decreases in transition region II, and spherical SiC particles are formed by deposition of the gas mixture. In border region III, the major substance of the white layer is the re-solidified SiO_2_. It is shown that the main ablation behavior at relatively higher temperature is the sublimation of the carbon fiber and SiC matrix, while the thermal decomposition and oxidation of the SiC matrix are the main ablation behaviors at relatively lower temperatures. The situation after irradiation with a sufficient number of pulses at different power densities is shown in Figure 7b-1,b-2, showing the effect of pulse number. With the increase of pulse number, the ablation pit is formed at a relatively high power density, and the transition region and border region diminish gradually.

## 4. Conclusions

In this paper, the surface morphology and ablation behavior of a C/SiC composite are studied. According to the microstructural observation, the effects of laser parameters and the laser ablation mechanism are analyzed. The main conclusions are as follows:

(1) The surface morphology of the C/SiC composite under laser irradiation usually includes three regions. In the center region, the compound composed of gases and vapors is generated by the sublimation of the SiC matrix and carbon fibers. The temperature decreases in transition region II, and spherical SiC particles are formed by the deposition of the gas mixture. In border region III, the major substance in the white layer is the re-solidified SiO_2_. It is shown that the main ablation behavior at relatively high temperatures is the sublimation of the carbon fibers and the SiC matrix, while thermal decomposition and oxidation of the SiC matrix are the main ablation behaviors at relatively lower temperatures.
(2) The power density plays an important role in the surface morphology after ablation. A clear boundary between the three regions will not take place until the power density is increased to a high enough value; the same is true for the formation of surface cracks. With the increase of laser power density, the ablation of the center region becomes more severe, and more spherical SiC particles can be found in the transition region.
(3) Furthermore, the pulse number also influences the ablation morphology significantly. As for the center region, when the power density is relatively low, even after being ablated for 100 pulses, the ablation damage is limited within the second layer under the sample surface. However, when the laser power density is relatively high and if the pulse number is larger than 50, an ablation pit takes place in the center region, and the transition region and border region diminish with the increase of pulse number.

The above research study on the effects of laser parameters and the laser ablation mechanism is helpful for material design and performance evaluation of C/SiC composites, and is of critical importance for laser machining processes of C/SiC composites.

## Figures and Tables

**Table 1:** The testing conditions for the laser ablation process.

Condition	Pulse Energy (J)	Peak Power (W)	Peak Power Density (W·cm^−2^)
A	1.5	1500	4.77 × 10^4^
B	2	2000	6.37 × 10^4^
C	2.5	2500	7.96 × 10^4^
D	3	3000	9.55 × 10^4^
E	3.5	3500	11.1 × 10^4^
F	3.8	3800	12.1 × 10^4^
